# Prognostic Factor Analysis for Risk‐Stratified Papillary Thyroid Carcinoma and Nomogram Development for Predicting Structural Incomplete Response to Radioactive Iodine Therapy in Intermediate‐Risk Patients

**DOI:** 10.1002/cnr2.70692

**Published:** 2026-09-16

**Authors:** Yamin Li, Zhaohui He, Min Zhao, Bin Zhang

**Affiliations:** ^1^ Department of Nuclear Medicine The First Affiliated Hospital of Soochow University Suzhou China

**Keywords:** LNM, LNR, nomogram, papillary thyroid carcinoma, prognosis, sTg

## Abstract

**Background:**

Structural incomplete response (SIR) after radioactive iodine (RAI) therapy indicates persistent or recurrent structural disease and has important implications for the long‐term management of patients with papillary thyroid carcinoma (PTC). However, predictors of SIR across recurrence‐risk strata remain incompletely characterized.

**Aims:**

To identify factors associated with SIR following RAI therapy in risk‐stratified PTC patients and to develop a predictive nomogram for intermediate‐risk patients.

**Methods and Results:**

We retrospectively analyzed 615 PTC patients who underwent thyroidectomy, lymph‐node dissection, and ^131^I therapy. Patients were re‐stratified according to the 2025 American Thyroid Association recurrence‐risk framework into a low/low‐to‐intermediate‐risk group and an intermediate‐high/high‐risk group. In 396 intermediate‐risk patients with lymph‐node metastasis, univariable and multivariable logistic regression analyses were used to identify independent predictors of SIR, and a nomogram was developed and internally validated. SIR rates differed significantly between risk groups (*p* < 0.001). Stimulated thyroglobulin (sTg) independently predicted SIR across all risk strata. The number of lymph‐node metastases (LNM) and lymph‐node yield additionally predicted SIR in intermediate‐high/high‐risk patients. In intermediate‐risk patients, age, tumor size, lymph‐node ratio, sTg, and LNM were independent predictors of SIR. The nomogram yielded an area under the curve of 0.865 (95% CI, 0.814–0.916) in the training cohort and 0.733 (95% CI, 0.617–0.849) in the internal hold‐out validation cohort; the bootstrap optimism‐corrected area under the curve was 0.853, and the optimism‐corrected calibration slope was 0.92.

**Conclusion:**

sTg and LNM are key predictors of SIR in risk‐stratified PTC patients. In this single‐center retrospective cohort, the nomogram showed good discrimination and calibration for SIR in intermediate‐risk patients; external multicenter validation is warranted before clinical application.

## 1 Introduction

According to the GLOBOCAN 2020 database from the World Health Organization's International Agency for Research on Cancer, thyroid cancer ranks ninth in global cancer incidence [1, 2]. It comprises three main histological categories: differentiated thyroid carcinoma (papillary, follicular, and oncocytic), medullary thyroid carcinoma (occasionally associated with multiple endocrine neoplasia Type 2) and anaplastic thyroid carcinoma, which usually arises from differentiated carcinoma and carries a high mortality. Although subtype distribution varies by country, papillary thyroid carcinoma (PTC) accounts for approximately 80% of cases. It is characterized by slow progression, low mortality, and generally favorable long‐term prognosis. However, it is frequently associated with lymph‐node metastasis. According to previous reports, LNM has been proven to be a risk factor for poor prognosis in thyroid cancer [3, 4]. In clinical practice, for low‐risk papillary thyroid carcinoma patients, the benefit of ^131^I ablation therapy in reducing tumor recurrence and mortality remains controversial. However, it has unique value in detecting occult metastatic lesions (especially microscopic lymph‐node metastases and distant metastases), which can provide important evidence for postoperative treatment decisions. In contrast, its value in intermediate‐high/high‐risk PTC is widely recognized. Reflecting the ongoing uncertainty in lower‐risk disease, recent trials have shown that RAI can be safely omitted in selected low‐risk patients [5], underscoring the need to individualize treatment.

The standard treatment of PTC is surgery followed, when indicated, by RAI therapy [6]. The 2025 ATA guidelines recommend a dynamic risk‐assessment system to customize treatment intensity and follow‐up, categorizing clinical treatment outcomes into four categories: excellent response (ER), indeterminate response (IDR), biochemical incomplete response (BIR), and structural incomplete response (SIR) [7]. SIR is defined as structural evidence of disease demonstrated by suspicious imaging findings or biopsy‐proven local or distant metastatic disease.

As a marker of disease progression, SIR importantly informs long‐term management and follow‐up. Although validated criteria based on age, histology, primary tumor size, surgery, and extrathyroidal extension (ETE) guide prognosis, the occurrence and determinants of SIR across different risk strata remain incompletely characterized. We therefore conducted a large retrospective study to examine the incidence and predictors of SIR after ^131^I therapy across risk strata and to build a nomogram for intermediate‐risk PTC—the group in which treatment decisions are most controversial and individualized risk assessment is most needed.

## 2 Materials and Methods

### 2.1 Initial Risk Stratification and Cohort Analysis

#### 2.1.1 Inclusion and Exclusion Criteria

This retrospective study was approved by the Ethics Committee (Institutional Review Board) of the First Affiliated Hospital of Soochow University (approval no. 2025003). Owing to the retrospective design using de‐identified data, the requirement for additional written informed consent was waived. The study was conducted in accordance with the Declaration of Helsinki. A retrospective collection and analysis of 615 PTC patients from the First Affiliated Hospital of Soochow University was conducted, including 189 males and 426 females, aged 16–80 years (42.05 ± 12.13 years).

##### 2.1.1.1 Inclusion Criteria

(1) Pathologically confirmed PTC; (2) First‐time ^131^I treatment postoperatively; (3) Regular oral levothyroxine therapy for thyroid stimulating hormone suppression after ^131^I treatment; (4) Follow‐up period greater than 6 months with complete medical records; (5) Patients with low/low‐to‐intermediate risk and intermediate‐high/high recurrence risk.

##### 2.1.1.2 Exclusion Criteria

(1) No postoperative ^131^I therapy; (2) Incomplete records or loss to follow‐up; and (3) Distant metastasis. Because patients with distant metastasis (M1) were excluded, ‘high risk’ in this cohort was defined solely by loco‐regional features (gross ETE/T4, extranodal extension, bulky nodal disease, aggressive histology, or gross residual disease); after exclusions, the combined intermediate‐high/high‐risk group comprised 318 patients (T3, *n* = 47; T4, *n* = 49).

#### 2.1.2 Study Methods

All patients underwent standardized thyroidectomy with systematic lymph‐node dissection and recurrent laryngeal nerve exploration guided by nodal status and local invasion. The total number of dissected nodes, number of positive nodes and presence of distant metastasis were recorded, and disease was staged by the AJCC/UICC 8th‐edition TNM system. When postoperative TSH exceeded 30 mU/L, sTg and thyroglobulin antibody (TgAb) were measured before ^131^I therapy. Treatment response was assessed 6 months after therapy using suppressed Tg, TgAb, diagnostic ^131^I whole‐body scintigraphy (^131^I‐Dx‐WBS) and SPECT/CT. The 6‐month time point was chosen because the ATA dynamic risk system evaluates response at 6–12 months and 6 months is a widely used early‐response assessment after the first RAI course. Patients were treated according to the ATA guidelines in force during the study period (2015 ATA) [6] and were re‐stratified for this analysis using the 2025 ATA recurrence‐risk framework [7]; risk tiers—low (< 10%), low‐to‐intermediate (10%–15%), intermediate‐high (16%–30%) and high (> 30%)—were defined by T category, extent/size of nodal disease, ETE, margins, histology and vascular invasion (full criteria in Figure S1). Responses were classified as ER, IDR, BIR or SIR; ER, IDR and BIR were grouped as non‐SIR for analysis (Figure S2).

#### 2.1.3 Statistical Methods

Analyses used IBM SPSS 27.0. Normally distributed data are presented as mean ± SD and non‐normally distributed data as median (P25, P75); categorical data as frequencies. Between‐group comparisons used the chi‐square or Fisher exact test; sTg and TgAb were compared with the Mann–Whitney U test. For significant continuous variables, ROC analysis determined optimal cutoffs for predicting SIR. Independent predictors were identified by multivariable logistic regression. All ^131^I activities are reported in millicuries (mCi), the unit used in clinical prescribing (1 mCi = 0.037 GBq). *p* < 0.05 was considered significant.

### 2.2 Patient Selection for Model Development

Based on the initial risk stratification results, only patients categorized as intermediate‐risk were eligible for subsequent model development.

#### 2.2.1 Inclusion Criteria for Model Development

(1) Patients with pathological diagnosis of PTC; (2) Patients with complete follow‐up data; (3) Age ≥ 18 years; (4) Patients who underwent total/near‐total thyroidectomy and lymph node dissection; (5) Patients classified as intermediate risk according to the 2025 ATA guidelines (combining patients with recurrence risk 10%–30%); (6) First‐time ^131^I treatment.

#### 2.2.2 Exclusion Criteria for Model Development

Exclusion criteria were as follows: (1) Patients with incomplete survival information; (2) Patients whose pathology after thyroidectomy and lymph node dissection showed no lymph node metastasis; (3) Patients with bone or distant metastases discovered before ^131^I treatment; (4) Patients with second primary malignant tumors.

#### 2.2.3 Treatment and Follow‐Up Strategy for Model Development

All patients underwent total or near‐total thyroidectomy with cervical lymph node dissection before the first ^131^I treatment. Patients were required to follow a low‐iodine diet 1 month before RAI treatment and during treatment, and discontinue levothyroxine tablets 3–4 weeks before RAI treatment. Before treatment, thyroid function tests, thyroid tumor markers, complete blood count, and biochemical indicators were examined to ensure all test results met treatment requirements before RAI treatment could be performed. The ^131^I dosage was determined based on postoperative pathological results, ultrasound examination, laboratory tests, and other imaging assessments, with stratified evaluation based on patient recurrence risk. After taking ^131^I, patients were instructed to hold vitamin C sublingually, drink plenty of water, and urinate frequently to promote radioactive iodine excretion, while receiving radiation isolation. All patients were required to take levothyroxine tablets regularly after RAI treatment to suppress TSH levels. Six months after treatment, treatment efficacy was evaluated based on serum suppressed thyroglobulin (Tg) and thyroglobulin antibody (TgAb) concentrations, combined with ^131^I‐Dx‐WBS and SPECT/CT imaging findings. Patient treatment responses were classified as excellent response (ER), indeterminate response (IDR), biochemical incomplete response (BIR), and structural incomplete response (SIR). This study grouped IDR, BIR and ER as the non‐SIR group for analysis.

#### 2.2.4 Statistical Methods for Model Development

Analyses used SPSS 27.0 and R 4.3.1. Patients were randomly partitioned 3:1 into training and validation sets using computer‐generated random numbers, independent of outcome. Optimal cutoffs for continuous variables were derived from ROC curves, and a nomogram was built from the independent predictors identified on multivariable analysis. Discrimination and calibration were assessed in both cohorts; internal validity was further examined by bootstrap resampling, and decision‐curve analysis (DCA) evaluated clinical utility. All tests were two‐sided, with *p* < 0.05 considered significant.

## 3 Results

### 3.1 Baseline Characteristics and Initial Risk Factor Analysis

#### 3.1.1 General Characteristics of All Patients (Table 1)

**TABLE 1:** Patient general information.

Clinicopathological characteristics	Low/low‐to‐intermediate Risk	Intermediate‐high/high Risk	Total	Percentage
Age (years)				
< 55	245 (48.32%)	262 (51.68%)	507	82.44%
≥ 55	52 (48.15%)	56 (51.85%)	108	17.56%
Gender				
Male	81 (42.86%)	108 (57.14%)	189	30.73%
Female	216 (50.70%)	210 (49.30%)	426	69.27%
T Stage				
T1	253 (61.41%)	159 (38.59%)	412	66.99%
T2	44 (41.12%)	63 (58.88%)	107	17.40%
T3	0 (0.0%)	47 (100.00%)	47	7.64%
T4	0 (0.0%)	49 (100.00%)	49	7.97%
N Stage				
N0	37 (92.50%)	3 (7.50%)	40	6.50%
N1	260 (45.22%)	315 (54.78%)	575	93.50%
^131^I Treatment dose (mCi)				
< 100	44 (100.00%)	0 (0.0%)	44	7.15%
100	141 (100.00%)	0 (0.0%)	141	22.93%
120	26 (100.00%)	0 (0.0%)	26	4.23%
150	86 (21.94%)	306 (78.06%)	392	63.74%
200	0 (0.0%)	12 (100.00%)	12	1.95%

#### 3.1.2 Efficacy Analysis of Low/Low‐To‐Intermediate Risk and Intermediate‐High/High Risk Patients

Statistical analysis showed significant differences in efficacy distribution among patients with different risk stratifications (*χ* ^2^ = 85.63, *p* < 0.001). Treatment efficacy evaluation results after initial ^131^I treatment showed that the SIR incidence rate in the low/low‐to‐intermediate risk group was 10.4%, significantly lower than the 38.1% in the intermediate‐high/high risk group (Table S1). This finding suggests that the overall response after treatment in the low/low‐to‐intermediate risk group was significantly better than that in the intermediate‐high/high risk group.

#### 3.1.3 Analysis of Factors Affecting Treatment Efficacy in Patients With Two Risk Stratifications

##### 3.1.3.1 Low and Low‐To‐Intermediate Risk Patients

In the low/low‐to‐intermediate risk group, there were no statistically significant differences in gender, T stage, LNY, LNR, and age (all *p* > 0.05); however, there were statistically significant differences in sTg levels, LNM, sTgAb, and ^131^I treatment dose (all *p* < 0.05) (Table 2).

**TABLE 2:** Clinical characteristic analysis of low/low‐to‐intermediate risk group patients.

Clinical characteristics	Non‐SIR	SIR	Test value	*p*
Gender			0.56	0.454
Male	72 (88.89%)	9 (11.11%)		
Female	194 (89.81%)	22 (10.19%)		
T Stage			1.23	0.267
T1	226 (83.33%)	27 (10.67%)		
T2	40 (90.91%)	4 (9.09%)		
LNM			—	0.02
> 0	229 (88.08%)	31 (11.92%)		
0	37 (100.00%)	0 (0.0%)		
LNY	14.25 (5, 19)	15.93 (5, 24)	3766^a^	0.43
LNR			0.0097	0.922
≥ 0.5	67 (90.54%)	7 (9.46%)		
< 0.5	199 (89.24%)	24 (10.76%)		
Age (years)			1.071	0.301
≥ 55	44 (84.62%)	8 (15.38%)		
< 55	222 (90.61%)	23 (9.39%)		
sTg (ng/mL)	10.7 (0.34, 11.58)	12.52 (0.13, 3.93)	11023.5^a^	0.007
sTgAb (ng/mL)	60.28 (10, 24.9)	136.99 (10.1, 137.25)	5052^a^	0.038
^131^I Treatment Dose (mCi)			—	< 0.001
150	86 (100.00%)	0 (0.0%)		
< 150	180 (85.31%)	31 (14.69%)		

^a^ Represents *U* values, “‐” indicates Fisher's exact test with only *p* values provided, the rest are *χ* ^2^ values; sTg refers to stimulated thyroglobulin before ^131^I treatment, TgAb refers to TgAb before ^131^I treatment.

**FIGURE 1:**
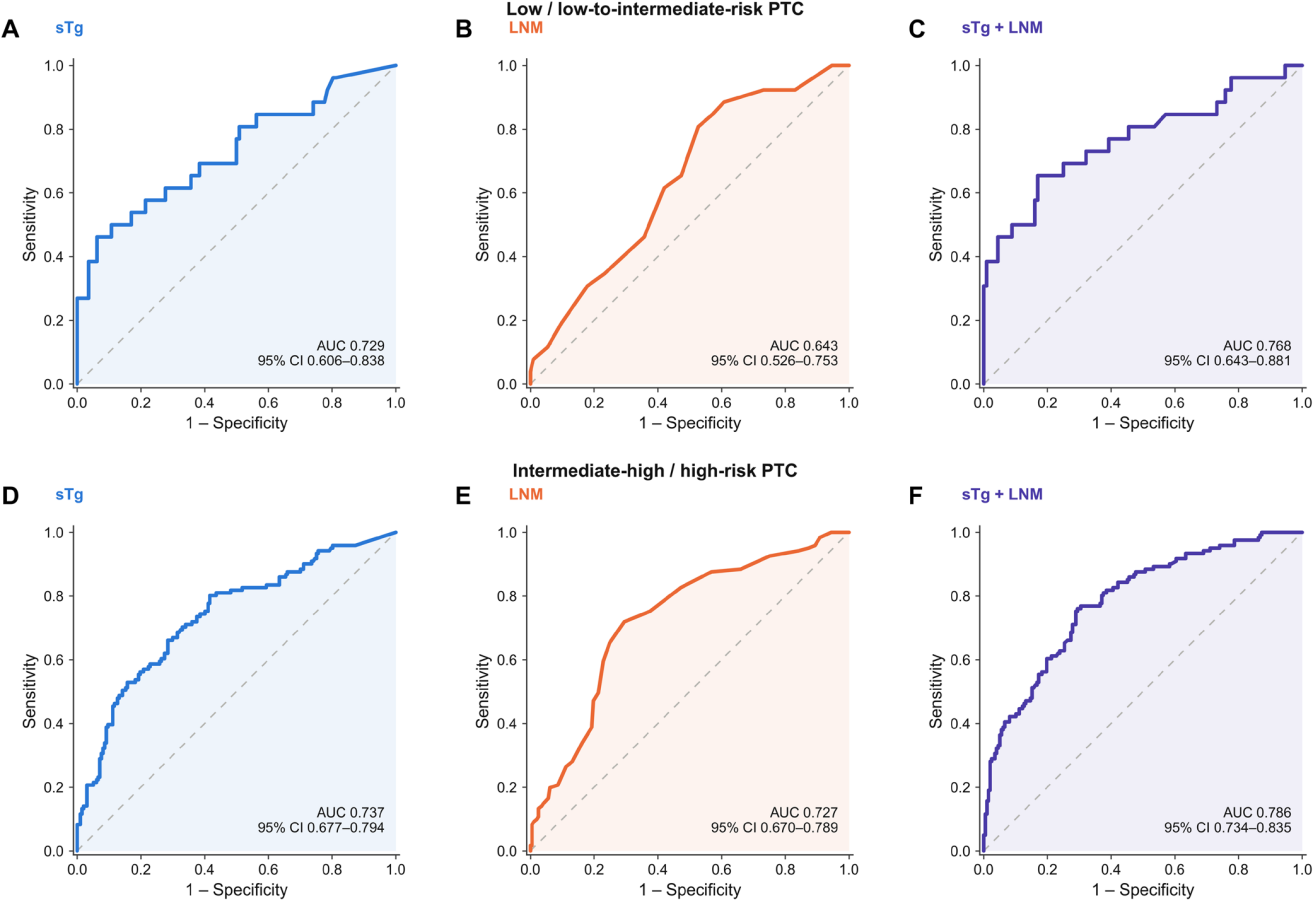
ROC curves for predicting SIR after ^131^I therapy, stratified by recurrence‐risk group. Top row (A–C), low/low‐to‐intermediate‐risk PTC; bottom row (D–F), intermediate‐high/high‐risk PTC. (A, D) stimulated thyroglobulin (sTg) alone; (B, E) number of lymph‐node metastases (LNM) alone; (C, F) sTg combined with LNM. The area under the curve (AUC) with its 95% confidence interval is shown in each panel; the dashed diagonal denotes the line of no discrimination. PTC, papillary thyroid carcinoma.

On ROC analysis, the optimal cutoffs for predicting SIR were 1.5 ng/mL for sTg (AUC 0.729, 95% CI 0.611–0.848) and 2.5 for LNM (AUC 0.643, 95% CI 0.533–0.753); the two combined reached an AUC of 0.768 (95% CI 0.652–0.883) (Figure 1A–C).

Multivariable analysis identified sTg, sTgAb, and ^131^I activity as factors associated with SIR in the low/low‐to‐intermediate‐risk group (Table S2).

##### 3.1.3.2 Intermediate‐High/High Risk Patients

The clinical characteristics of intermediate‐high/high risk patients are shown in Table 3. The analysis results showed statistically significant differences in sTg, LNM, LNY, LNR, TgAb and ^131^I treatment dose.

**TABLE 3:** Clinical characteristic analysis of intermediate‐high/high risk group patients.

Clinical characteristics	Non‐SIR	SIR	Test value	*p*
Gender			1.1545	0.2826
Male	62 (57.41%)	46 (42.59%)		
Female	135 (64.29%)	75 (35.71%)		
T Stage			2.0841	0.5551
T1	98 (61.64%)	61 (38.36%)		
T2	35 (55.56%)	28 (44.44%)		
T3	32 (68.09%)	15 (31.91%)		
T4	32 (65.31%)	17 (34.69%)		
LNM	10.51 (6.75, 12.25)	16.38 (11, 20)	6509^a^	< 0.001
LNY	29.72 (14, 40.25)	36.23 (20.75, 46)	9540.5^a^	0.0028
LNR			9.07	0.0026
≥ 0.5	63 (51.22%)	60 (48.78%)		
< 0.5	134 (68.72%)	61 (31.28%)		
Age (years)			2.13	0.145
≥ 55	40 (71.43%)	16 (28.57%)		
< 55	157 (59.92%)	105 (40.08%)		
sTg (ng/mL)	27.31 (0.57, 25.25)	157.18 (13.58, 126.95)	6262^a^	< 0.001
TgAb (ng/mL)	180.15 (12.75, 51.95)	136.9 (11.6, 18.75)	13772^a^	0.0197
^131^I Treatment Dose (mCi)			5.69	0.017
200	3 (25.00%)	9 (75.00%)		
150	194 (63.40%)	112 (36.60%)		

^a^ Represents *U* values, the rest are *χ* ^2^ values.

Optimal cutoffs were 11.625 ng/mL for sTg (AUC 0.737, 95% CI 0.680–0.795) and 11.5 for LNM (AUC 0.727, 95% CI 0.670–0.789), with a combined AUC of 0.786 (95% CI 0.734–0.835) (Figure 1D–F).

Multivariate logistic regression analysis was performed with statistically significant factors from univariate analysis (sTg, LNM, LNY, LNR, TgAb, and ^131^I treatment dose) as independent variables and SIR as the dependent variable. The results are shown in Table 4. sTg, LNM, and LNY were independent predictive factors for SIR in intermediate‐high/high‐risk patients.

**TABLE 4:** SIR multivariate logistic regression analysis.

Clinical Characteristics	*β*	S.E.	*Z*	*p*	OR (95% CI)
^131^I Treatment Dose (mCi)					
150					1.00 (Reference)
200	0.62	0.82	0.76	0.445	1.87 (0.38~9.25)
LNM	0.21	0.04	5	< 0.001	1.23 (1.13~1.34)
LNY	−0.04	0.01	−2.85	0.004	0.96 (0.93~0.99)
sTg	0.01	0	4.08	< 0.001	1.01 (1.01~1.01)
TgAb	0	0	0.34	0.737	1.00 (1.00~1.00)
LNR	−1.14	0.97	−1.18	0.24	0.32 (0.05~2.14)

### 3.2 Predictive Model Development in Intermediate‐Risk Patients

#### 3.2.1 Patient Clinical Characteristics and Outcomes

This study included a total of 396 PTC patients. The clinicopathological characteristics of patients in the training and validation groups are shown in Table 5. There were no statistically significant differences in clinical data between the two groups.

**TABLE 5:** Clinical and pathological characteristics of patients in training and validation groups.

Clinical characteristics	Training group (*n* = 297)	Validation group (*n* = 99)	*p*
Age	41.85 ± 11.97	42.39 ± 11.78	0.693
Gender			0.437
Female	207 (69.7%)	73 (73.7%)	
Male	90 (30.3%)	26 (26.3%)	
Tumor size (cm)	1.69 ± 0.97	1.82 ± 1.04	0.289
≤ 1	83 (27.9%)	24 (24.2%)	
> 1	214 (72.1%)	75 (75.8%)	
Tumor range			0.256
Unilateral	133 (44.8%)	49 (49.5%)	
Bilateral	164 (55.2%)	50 (50.5%)	
Tumor focality			0.533
Single	100 (33.7%)	30 (30.3%)	
Multiple	197 (66.3%)	69 (69.7%)	
LNM	8.26 ± 5.91	8.27 ± 6.99	0.982
LNY	24.95 ± 16.81	28.14 ± 20.55	0.166
LNR	0.39 ± 0.23	0.37 ± 0.23	0.472
sTg (ng/mL)	12.13 ± 21.22	11.44 ± 19.67	0.77
TgAb (IU/mL)	113.57 ± 399.15	133.28 ± 441.71	0.669
T Stage			0.737
T1	201 (67.7%)	64 (64.6%)	
T2	48 (16.2%)	17 (17.2%)	
T3	23 (7.7%)	11 (11.1%)	
T4	25 (8.4%)	7 (7.1%)	
ETE			0.816
Absent	130 (43.8%)	42 (42.4%)	
Present	167 (56.2%)	57 (57.6%)	
RAI treatment dose (mCi)	131.99 ± 24.04	130.81 ± 24.44	0.677
Efficacy			0.311
SIR	60 (20.2%)	25 (25.3%)	
Non‐SIR	237 (79.8%)	74 (74.7%)	

#### 3.2.2 Logistic Regression Analysis

3.2.2

In the training cohort, age [OR (95% CI): 1.03 (1.01–1.05), *p* = 0.026], tumor size [(≤ 1 vs. > 1), OR (95% CI): 3.57 (1.55–8.23), *p* = 0.003], LNM [OR (95% CI): 1.13 (1.07–1.18), *p* < 0.001], LNR [OR (95% CI): 25.20 (7.15–88.85), *p* < 0.001], and sTg [OR (95% CI): 1.03 (1.02–1.05), *p* < 0.001] were determined to have significant correlations through univariate analysis. After multivariate analysis, these 5 variables—age, LNM, sTg, tumor size, and LNR—ultimately became independent predictive factors for SIR (Table 6).

**TABLE 6:** Univariate and multivariate analysis in training group.

Variable	Univariate					Multivariate				
	*β*	S.E	*Z*	*p*	OR (95% CI)	*β*	S.E	*Z*	*p*	OR (95% CI)
Age	0.03	0.01	2.22	0.026	1.03 (1.01~1.05)	0.04	0.01	2.47	0.009	1.04 (1.01~1.07)
Gender										
Female					1.00					
Male	0.27	0.31	0.88	0.376	1.31 (0.72~2.39)					
Tumor Size (cm)										
≤ 1					1.00					1.00
> 1	1.27	0.43	2.99	0.003	3.57 (1.55~8.23)	1.03	0.45	2.27	0.023	2.81 (1.15~6.84)
Tumor range										
Unilateral					1.00					
Bilateral	0.42	0.30	1.41	0.159	1.52 (0.85~2.73)					
Tumor focality										
Single					1.00					
Multiple	0.41	0.32	1.28	0.201	1.51 (0.80~2.84)					
LNM	0.12	0.03	4.50	< 0.001	1.13 (1.07~1.18)	0.12	0.03	3.80	< 0.001	1.12 (1.06~1.19)
LNY	0.00	0.01	0.53	0.594	1.00 (0.99~1.02)					
LNR	3.23	0.64	5.02	< 0.001	25.2 (7.15~88.85)	2.96	0.73	4.07	< 0.001	19.37 (4.62~80.57)
sTg (ng/mL)	0.03	0.01	4.30	< 0.001	1.03 (1.02~1.05)	0.03	0.01	2.98	0.003	1.03 (1.01~1.04)
TSH (ng/mL)	0.01	0.01	1.00	0.315	0.99 (0.98~1.01)					
TgAb (IU/mL)	0.00	0.00	1.62	0.105	1.00 (0.99~1.00)					
T Stage										
T1					1.00					
T2	0.21	0.39	0.55	0.586	1.23 (0.58~2.64)					
T3	0.13	0.58	0.23	0.817	0.87 (0.28~2.72)					
T4	0.27	0.50	0.54	0.588	1.31 (0.49~3.50)					
ETE										
Absent					1.00					
Present	0.19	0.29	0.66	0.510	1.21 (0.68~2.16)					
RAI Treatment Dose (mCi)										
100					1.00					
150	0.15	0.31	0.49	0.627	1.16 (0.64~2.11)					

#### 3.2.3 ROC Analysis

ROC‐derived cutoffs were age 42.5 years, sTg 7.46 ng/mL, LNM 8.5, LNR 0.30 and tumor size 1.05 cm (Table S3). These thresholds were used only to construct the point‐based nomogram; the age categories in Table 1 (< 55 vs. ≥ 55 years) are descriptive (per AJCC staging) and differ from the model cutoff of 42.5 years, and the continuous‐variable regression is reported in Table 6 [8].

#### 3.2.4 Establishment of Nomogram Model

**FIGURE 2:**
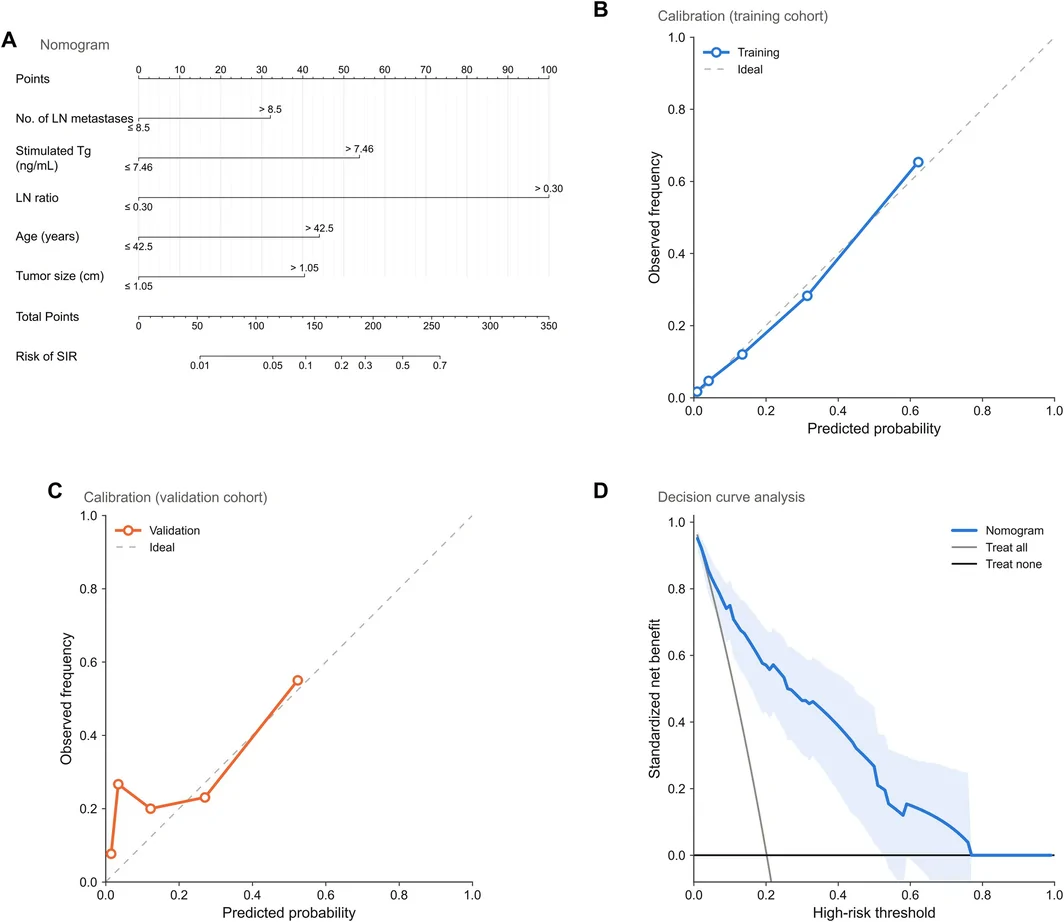
Nomogram for predicting SIR after ^131^I therapy in intermediate‐risk PTC, and its performance. (A) Nomogram incorporating five predictors—number of lymph‐node metastases (> 8.5), stimulated thyroglobulin (> 7.46 ng/mL), lymph‐node ratio (> 0.30), age (> 42.5 years) and tumor size (> 1.05 cm). For each predictor, draw a vertical line to the Points axis to obtain its score; sum the scores to obtain Total Points, then read the corresponding Risk of SIR on the bottom axis. (B, C) Calibration curves in the training (B) and validation (C) cohorts; the dashed line represents ideal calibration and the solid line the nomogram. (D) Decision curve analysis showing the standardized net benefit of the nomogram (blue) across high‐risk threshold probabilities, compared with the treat‐all (grey) and treat‐none (black) strategies; the shaded band is the 95% confidence interval. PTC, papillary thyroid carcinoma.

To predict the risk of SIR occurrence in intermediate‐risk PTC patients, these independent variables were extracted from the training cohort to establish a nomogram model (Figure 2A). In the prediction model, for the variable “LNM” “1” indicates that the total number of LNM is greater than 8.5; for the variable “sTg” “1” indicates that the sTg level is greater than 7.46 ng/mL; for the variable “age” “1” indicates age greater than 42.5 years; for the variable “LNR” “1” indicates that the LNM/LNY ratio is greater than 0.30; for the variable “tumor size” “1” indicates tumor size greater than 1.05 cm. Each variable is assigned a score based on its impact on the outcome event. Scores are calculated by drawing a line perpendicular to each risk factor axis until it intersects with the top line labeled “Points”. Then, the scores for all risk factors are summed. Finally, a line is drawn downward from the “Total Points” axis to determine the total score. According to the total score of each factor in the chart, the higher the total score, the greater the likelihood that the patient will develop SIR after ^131^I treatment.

#### 3.2.5 Model Validation

Calibration curves showed good agreement between predicted and observed SIR in both cohorts (Figure 2B,C). The AUC of the nomogram for predicting SIR was 0.865 (Table S4). With 60 SIR events and five predictors, the events‐per‐variable ratio was 12 [9]. Bootstrap internal validation (1000 resamples with Harrell optimism correction) yielded an optimism‐corrected C‐statistic of 0.853 (apparent 0.865; optimism 0.011) and an optimism‐corrected calibration slope of 0.92, indicating minimal in‐sample overfitting [10]. All variance inflation factors were < 1.1, and the dichotomized LNM (> 8.5) and LNR (> 0.30) were only weakly correlated (phi ~0.27), supporting retention of both variables.

The DCA decision curve (Figure 2D) showed that the model's decision curve had a net benefit (NB) higher than the two ineffective lines within the threshold probability range of 1%–95%.

## 4 Discussion

We stratified patients into low/low‐to‐intermediate risk and intermediate‐high/high risk groups based on the clinical pathological characteristics of PTC patients after initial treatment (total/near‐total thyroidectomy + lymph node dissection), using the 2025 ATA recurrence risk stratification system. After ^131^I treatment, combined with follow‐up data including serological indicators (Tg, TgAb, TSH levels) and imaging examinations (neck ultrasound, diagnostic whole‐body scintigraphy) at 6 months post‐treatment, patient treatment responses were classified into two different types: SIR and non‐SIR. Further analysis revealed that factors affecting patient treatment outcomes differed within different risk groups.

In this study, sTg level was an independent predictive factor for SIR occurrence in patients with different risk stratifications, while LNM and LNY were independent predictive factors for SIR in intermediate‐high/high risk patients. ROC curve analysis showed that the optimal cutoff values for sTg combined with LNM for predicting SIR in intermediate‐high/high risk patients were 11.625 ng/mL and 11.5, respectively. The area under the curve was 0.786, indicating high accuracy, and this result is consistent with recent findings from other studies. Past research has shown that approximately 20%–90% of PTC patients develop lymph node metastases [11, 12]. Cervical lymph node metastases, such as central lymph node metastases and lateral lymph node metastases, have been proven to be factors affecting poor prognosis in PTC. Patients with lymph node metastases have increased recurrence risk and decreased survival rates [13, 14]. These studies indicate that lymph node metastases predict higher recurrence rates and poorer prognosis, which is consistent with our study results.

The number of metastatic lymph nodes is an important parameter in the ATA risk stratification system for predicting PTC recurrence. However, its predictive value for initial treatment response in certain specific patients remains to be determined. Sung et al. reported that for Stage I PTC patients under 45 years of age, the number of metastatic lymph nodes may be a potentially important risk factor for poor initial treatment response [15]. This finding emphasizes the potential value of lymph node metastases in prognostic assessment of young, low‐risk PTC patients. However, in our study, we found that the number of metastatic lymph nodes was only an independent predictive factor for poor prognosis in intermediate‐high/high risk patients, while it did not show independent predictive value in low/low‐to‐intermediate risk patients.

This difference may be attributed to two factors. First, the inclusion criteria for the lower‐risk group were broader than in earlier studies, producing greater heterogeneity. Second, lower‐risk patients received more stratified activities of 30–150 mCi, whereas higher‐risk patients received standardized higher activities (150–200 mCi); this difference in intensity, together with a greater proportion of microscopic, iodine‐avid disease in lower‐risk patients, may have reduced the prognostic value of LNM. Because administered ^131^I activity is largely determined by recurrence‐risk category, it partly reflects disease severity (confounding by indication) rather than an independent cause; consistent with this, activity was not an independent predictor of SIR in the intermediate‐risk model.

In recent years, with in‐depth research on the biological behavior and disease progression mechanisms of PTC, the clinical application value of sTg has been further expanded. Some scholars have proposed that serum sTg levels can not only serve as a sensitive indicator for monitoring disease recurrence or progression during postoperative follow‐up, but may also become an important reference for disease risk stratification. This perspective provides new research directions for individualized management and precision treatment of PTC. Serum Tg is mainly produced by thyroid tissue or its metastatic sites; therefore, sTg levels can serve as an important tumor marker for evaluating whether postoperative patients have residual lesions or recurrent disease. Postoperatively, changes in sTg levels in patients reflect the cellular activity of thyroid or metastatic lesions and the residual tumor burden. If sTg levels continue to rise, it may indicate that patients have postoperative residual lesions or disease recurrence [16]. Some studies have shown that there is a correlation between sTg levels and structural incomplete response: when sTg levels are below 1 ng/mL, the incidence of structural incomplete response is 0%; when sTg levels are between 1‐10 ng/mL, the incidence of structural incomplete response is 1.73%; when sTg levels are above 10 ng/mL, structural incomplete response significantly increases to 42.64% [17]. In our study, we observed findings closely related to the aforementioned results. The correlation between sTg levels and SIR also showed a similar pattern, further confirming the effectiveness of these data in evaluating treatment outcomes. Therefore, the higher the sTg level, the greater the likelihood that patients will experience poor prognosis.

Furthermore, Wang et al. proposed the correlation between Tg levels and LNM [18]. On one hand, LNM is very common in PTC. Combined with this study and previous research results, LNM is considered an independent predictive factor for poor prognosis in thyroid cancer. On the other hand, elevated serum Tg levels are usually associated with thyroid tissue stimulation or increased tumor burden. Therefore, both LNM and elevated Tg levels can serve as important indicators of tumor progression, reflecting disease deterioration trends and suggesting that patients may face greater treatment challenges and recurrence risks. The impact of lymph node metastasis on survival prognosis in PTC patients remains somewhat controversial, mainly because multiple studies have failed to demonstrate a clear statistical association between lymph node metastasis status and patients' long‐term survival [19, 20]. Although the impact of lymph node metastasis on overall survival rates in PTC patients remains controversial, its correlation with increased risk of death and recurrence does highlight the importance of lymph node status in prognostic assessment. In the AJCC 8th edition staging system, for PTC patients over 55 years old, staging adjustments are made if there is cervical lymph node metastasis. Particularly if the tumor has spread to lymph nodes, the stage may be upgraded from Stage I to a higher stage [21]. Wang et al. proposed that in PTC patients under 55 years old, lymph node metastasis significantly affected disease‐free survival [18]. In line with this, our study further found that the number of metastatic lymph nodes is an important predictive factor for structural incomplete response after ^131^I treatment in intermediate‐risk PTC patients. The number of metastatic lymph nodes should be considered as a factor in treatment and follow‐up decisions for intermediate‐risk PTC patients.

However, in clinical practice, whether there are significant differences in recurrence risk and long‐term prognosis between patients with only 1 metastatic lymph node found among 10 resected lymph nodes versus patients with 9 positive nodes among 10 resected lymph nodes remains insufficiently elucidated. LNR is regarded as a quantitative indicator of the extent of metastatic disease after cervical lymph node dissection. Both a higher number of positive lymph nodes or resection of only a small number of lymph nodes may lead to elevated LNR. Conversely, when more total lymph nodes are resected, the same number of positive lymph nodes would yield a lower LNR. It is important to understand that LNR not only reflects disease burden but also reflects the extent of cervical lymph node dissection and the thoroughness of pathological analysis. Research has shown that LNR values, as a prognostic indicator, play an important role in survival prediction for PTC patients [22]. Specifically, elevated LNR values may indicate higher tumor burden in patients, suggesting increased likelihood of lymph node metastasis, thereby affecting disease prognosis. The elevation of this indicator is closely related to shortened patient survival, showing negative correlation in both disease‐specific survival and overall survival rates. Therefore, LNR provides a more nuanced measurement than simply counting positive lymph nodes or using TNM system lymph node classification, as it simultaneously considers the extent of surgical intervention and the precision of pathological assessment.

Many studies have shown that higher LNR is associated with poorer disease prognosis. Hei et al.'s research team proposed an important viewpoint in their study: PTC patients with higher LNR may be more prone to developing SIR and exhibit a higher risk of disease persistence or are more likely to experience disease recurrence [22]. This finding provides a new perspective for understanding the relationship between lymph node metastatic burden and clinical prognosis. Vas Nunes et al. reported that LNR is an important independent prognostic factor for PTC, capable of effectively combining with existing TNM staging systems and ATA risk stratification frameworks to improve the accuracy of prognostic assessment. The clinically relevant cutoff point they proposed was 0.3, indicating 1 positive lymph node among 3 lymph nodes [23]. Our study results are consistent with these research findings. In intermediate‐risk PTC patients, our study observed that patients with elevated LNR levels are more prone to developing SIR. The critical LNR threshold for predicting SIR was 0.30 (AUC 0.74; sensitivity 50%, specificity 93%).

Beyond LNM and sTg, older age (associated with reduced radioiodine avidity), larger tumor size, and higher LNR each contributed independently to SIR. The biological determinants of RAI response were not captured here: BRAF V600E downregulates iodine‐handling genes including the sodium‐iodide symporter (NIS), reducing radioiodine avidity [24], and MAPK‐pathway inhibition can restore iodine uptake in refractory disease [25], while BRAF/TERT status and proliferation indices such as Ki‐67 influence aggressiveness and outcome [26]. Incorporating such markers and tumor thyroglobulin expression into future nomograms is an important direction.

We developed a nomogram from 297 training patients and validated it in 99 patients. Several limitations should be noted. First, the retrospective, single‐center design may introduce selection bias and limits generalizability; multicenter data are needed. Second, we deliberately used the structural endpoint (SIR) rather than the biochemical one because BIR may reclassify to ER over time; nonetheless, early SIR may reflect occult residual nodal disease missed at initial surgery as well as true recurrence, and longer follow‐up is warranted. Third, continuous predictors were dichotomized to yield a bedside‐usable score, which discards information and may reduce reproducibility; we therefore also report the continuous‐variable model and bootstrap‐corrected performance. Fourth, although discrimination fell from 0.865 (training) to 0.733 (hold‐out), the confidence intervals overlap and the optimism‐corrected estimate (0.853) indicates limited overfitting; external validation and recalibration remain necessary. Finally, our cohort comprises patients selected for and treated with RAI, so the nomogram predicts response within this population rather than informing the decision to administer RAI; whether similar predictors apply to an RAI‐naive intermediate‐risk cohort warrants dedicated study. Although the individual predictors are established, the contribution of this work lies in comparing SIR determinants across ATA risk strata and integrating them into a calibrated, decision‐analytically validated nomogram for the intermediate‐risk “grey zone.”

## 5 Conclusions

sTg and LNM were independent predictive factors for SIR in intermediate‐high/high risk PTC in both univariate and multivariate analyses. We further developed a nomogram that showed good discrimination and calibration for SIR in intermediate‐risk PTC; given the single‐center, retrospective design, external multicenter validation is needed before routine clinical use.

## Author Contributions

**Yamin Li:** conceptualization, methodology, investigation, validation, writing – review and editing, writing – original draft, software, data curation. **Zhaohui He:** data curation, validation. **Min Zhao:** software, data curation. **Bin Zhang:** writing – original draft, writing – review and editing, supervision, project administration.

## Funding

The authors have nothing to report.

## Consent

The requirement for written informed consent was waived by the Ethics Committee of the First Affiliated Hospital of Soochow University (approval no. 2025003) owing to the retrospective design and the use of de‐identified data.

## Conflicts of Interest

The authors declare no conflicts of interest.

## Supporting information


**Figure S1:** Risk stratification for recurrence in patients with PTC.
**Figure S2:** Thyroid cancer treatment response assessment in 2025 ATA guidelines.

**Table S1:** Patient efficacy analysis.
**Table S2:** Multivariate logistic regression analysis.
**Table S3:** ROC curve analysis.
**Table S4:** Area under the curve for predicting SIR.

## Data Availability

The data that support the findings of this study are available on request from the corresponding author. The data are not publicly available due to privacy or ethical restrictions.
