# Supplementary material for: Prognostic Factor Analysis for Risk‐Stratified Papillary Thyroid Carcinoma and Nomogram Development for Predicting Structural Incomplete Response to Radioactive Iodine Therapy in Intermediate‐Risk Patients
Source: Cancer Rep (Hoboken). 2026 Sep 16;9(9):e70692. doi: 10.1002/cnr2.70692 (PMC13581434; doi:10.1002/cnr2.70692)
Supplement: Supplementary file 1 [file CNR2-9-e70692-s001.docx]

**Supplementary Material 2**

**Table S1** Patient Efficacy Analysis

| **GROUP** | **Non-SIR** | **SIR** | **χ^2^** | ***P*** |
| --- | --- | --- | --- | --- |
| Low and low-to-intermediate Risk | 89.6% (266/297) | 10.4% (31/297) |  |  |
| Intermediate-high and high Risk | 61.9% (197/318) | 38.1% (121/318) | 85.63 | ＜0.001 |
| Total | 75.3% (463/615) | 24.7% (152/615) |  |  |

**Table S2** Multivariate Logistic Regression Analysis

| **Clinical Characteristics** | **β** | **S.E.** | **Z** | ***P*** | **OR (95% CI)** |
| --- | --- | --- | --- | --- | --- |
| sTg | 0.02 | 0.01 | 2.04 | 0.042 | 1.02 (1.01~1.04) |
| TgAb | 0.01 | 0 | 2.35 | 0.019 | 1.01 (1.01~1.01) |
| ^131^I Treatment Dose | -0.05 | 0.01 | -4.64 | <0.001 | 0.96 (0.94~0.97) |
| LNM | 0.24 | 0.13 | 1.81 | 0.07 | 1.27 (0.98~1.65) |

**Table S3** ROC Curve Analysis

| **Variable** | **AUC** | **95% CI** | **Sensitivity** | **Specificity** | **Cutoff Value** | ***P*** |
| --- | --- | --- | --- | --- | --- | --- |
| Age | 0.6 | 0.52-0.68 | 0.62 | 0.62 | 42.5 | 0.026 |
| sTg (ng/mL) | 0.72 | 0.64-0.79 | 0.71 | 0.67 | 7.46 | 0.003 |
| LNM | 0.66 | 0.58-0.67 | 0.59 | 0.68 | 8.5 | <0.001 |
| LNR | 0.74 | 0.67-0.80 | 0.5 | 0.93 | 0.3 | <0.001 |
| Tumor Size (cm) | 0.61 | 0.53-0.68 | 0.32 | 0.88 | 1.05 | 0.023 |

**Table S4** Area Under the Curve for Predicting SIR

|  | Training Group | |  | Validation Group | |
| --- | --- | --- | --- | --- | --- |
|  | AUC | 95% CI |  | AUC | 95% CI |
| Nomogram | 0.865 | 0.814-0.916 |  | 0.733 | 0.617-0.849 |
