# Supplementary material for: Prognostic Factor Analysis for Risk‐Stratified Papillary Thyroid Carcinoma and Nomogram Development for Predicting Structural Incomplete Response to Radioactive Iodine Therapy in Intermediate‐Risk Patients
Source: Cancer Rep (Hoboken). 2026 Sep 16;9(9):e70692. doi: 10.1002/cnr2.70692 (PMC13581434; doi:10.1002/cnr2.70692)
Supplement: Supplementary file 2 [file CNR2-9-e70692-s002.docx]

**Supplementary Material 1**

**Figure S1.** Risk Stratification for Recurrence in Patients with PTC

**Figure S2.** Thyroid Cancer Treatment Response Assessment in 2025 ATA Guidelines
